# Causative agents and outcome of spontaneous bacterial peritonitis in cirrhotic patients: community-acquired versus nosocomial infections

**DOI:** 10.1186/s12879-019-4102-4

**Published:** 2019-05-23

**Authors:** Xiurong Ding, Yanhua Yu, Ming Chen, Chen Wang, Yanfang Kang, Jinli Lou

**Affiliations:** Department of Clinical Laboratory, Beijing Youan Hospital, Capital Medical University, Beijing, 100069 China

**Keywords:** Liver cirrhosis, Community-acquired, Nosocomial, Spontaneous bacterial peritonitis, Outcome

## Abstract

**Background:**

Spontaneous bacterial peritonitis (SBP) is a serious complication and common cause of death in patients with liver cirrhosis. This study was conducted to compare the microbiological characteristics, drug resistance, and treatment outcomes for nosocomial SBP and community-acquired SBP.

**Methods:**

A retrospective study was performed on 334 patients with culture-positive SBP at Beijing Youan Hospital, China, between January 2012 and December 2016. The medical records for these patients were reviewed, and their clinical and laboratory data were analyzed.

**Results:**

A total of 155 (46.4%) patients with nosocomial SBP and 179 (53.6%) with community-acquired SBP were included in this study. From the patients’ ascitic fluids, 334 pathogenic strains, including 178 Gram-negative bacterial strains, 138 Gram-positive bacterial strains and 18 other microbial strains were isolated. *E. coli* was the major pathogen (24.3%), followed by *Klebsiella pneumoniae* (12.0%) and *Enterococcus faecium* (10.5%). The proportion of *Enterococcus* was significantly higher in the patients with nosocomial SBP (6.1% vs. 27.7%, *P* < 0.001) than in the patients with community-acquired SBP. The main pathogens isolated from the nosocomial infections were significantly more resistant to the first-line recommended drug. Compared with community-acquired SBP, nosocomial SBP had a poorer outcome (36.8% vs. 24.6%; *P* = 0.016). The independent predictors for 30-day mortality included nosocomial infection, Child-Pugh classification, hepatocellular carcinoma, renal failure and hepatic encephalopathy.

**Conclusion:**

Gram-negative bacteria were the major pathogens involved in SBP in the cirrhotic patients. The strains isolated from the patients with nosocomial SBP displayed higher drug resistance than those isolated from patients with community-acquired SBP. Compared with community-acquired SBP, nosocomial SBP had a poorer outcome. When choosing drug treatments, the acquisition site of infection and the local epidemiological situation should be taken into account.

## Background

Spontaneous bacterial peritonitis (SBP) is a well-recognized and prevalent complication seen in cirrhotic patients with ascites, occurring in 10–25% of these patients [1–4]. It leads to more severe liver function damage, multi-organ failure and sepsis, thus affecting the prognosis of such patients [5–7]. Once SBP is diagnosed, appropriate antibiotic treatment must be started as soon as possible, without prior knowledge of the causative organisms or their in vitro drug sensitivities [1, 2]. Because Gram-negative *Enterobacteriaceae* members are recognized as the most common causative organisms of SBP, third-generation cephalosporins (TGCs) have been recommended as the first-line therapies for this condition [1, 2, 8]. However, as cirrhotic patients require frequent hospitalization, undergo numerous invasive procedures, and are subject to antibiotic prophylaxis, some studies have suggested that the bacterial spectrum and resistance profiles of the causative pathogens of SBP have changed and show huge regional variation [4, 9, 10]. Thus, the latest guidelines from the European Association for the Study of Liver (EASL) recommend that for empirical treatment of SBP in cirrhotic patients with ascites, distinguishing nosocomial SBP from community-acquired SBP is necessary [11]. Moreover, whether the infection site is community-acquired or nosocomically acquired will influence the clinical outcome for patients with SBP, but large differences exist in the data from different studies [4, 12, 13].

This study aimed to determine whether differences exist between the clinical and microbiological characteristics of nosocomial and community-acquired SBP. We also explored the use of a comprehensive approach to determine the possible prognostic factors for hospital mortality in relation to SBP.

## Methods

### Patients and study design

The medical records of 334 patients (> 18 years) with culture-positive SBP and who were hospitalized at Beijing Youan Hospital, Capital Medical University (Beijing Institute of Hepatology) from January 2012 to December 2016 were reviewed. We only included one episode of SBP for each patient within the study period. Patients with a culture that was positive for highly suspicious skin contaminants, namely, *coagulase negative Staphylococci* (when the same strain was isolated twice or more from one patient and the treatment records were available, it was counted in this study), *Corynebacterium*, *Propionibacterium*, or *Bacillus* spp., and those with secondary peritonitis were excluded from our study. This study was carried out in accordance with the principles of the Declaration of Helsinki and was formally approved by the Institutional Medical Ethics Committee of Beijing Youan Hospital, Capital Medical University, China.

### Definitions

Culture-positive SBP was defined as a sample having a polymorphonuclear leukocyte count of ≥250 cells per mm^3^ in ascitic fluid and an ascitic fluid culture that was positive for a single organism. Community-acquired SBP was defined as an infection diagnosed within the first 48 h of admission to hospital, whereas a diagnosis made more than 48 h after hospitalization was defined as nosocomial SBP. Secondary peritonitis was considered in patients where the following applied: a polymicrobial infection, peritoneal dialysis, an indwelling abdominal catheter, and a recent history of abdominal surgery. The baseline data of patients with nosocomial SBP were assessed at the moment of SBP diagnosis. By reviewing the medical records and implementing a telephone follow-up survey, each patient was followed for 30 d after their diagnosis of SBP to determine the outcome.

### Laboratory testing

Bacterial identification and antibiotic susceptibility tests were performed according to the standard procedures established by the Clinical and Laboratory Standards Institute (CLSI). Cultivated microorganisms were identified using PHOENIX-100 (Becton Dickinson, Mountain View, CA, United States) instrumentation and matrix-assisted laser desorption/ionization-time of flight mass spectrometry (Bruker Daltonics, Germany). The antibiotic susceptibility testing was performed using PHOENIX-100 and confirmed by the Kirby-Bauer method on Müller-Hinton agar (Oxoid, Basingstoke, UK). The drug sensitivity test results were assessed according to the CLSI standards of 2012. *Staphylococcus aureus* ATCC25923, *Escherichia coli* ATCC25922, and *Pseudomonas aeruginosa* ATCC27853 were used as the quality control strains.

### Statistical analysis

SPSS 17.0 (SPSS, Chicago, IL, USA) was used for all the statistical analyses. Comparisons of the categorical variables were performed using χ^2^, Fisher’s exact test, or continuity correction, as appropriate. Continuous variables were compared using the Student’s *t* test. To determine the risk factors associated with mortality, the proportional-hazards Cox regression model was used to control the effects of the confounding variables. Variables with *P <* 0.05 in the univariate analyses were candidates for multivariate analysis. All *P* values were 2-tailed, and *P* values of *<* 0.05 were considered to be statistically significant.

## Results

### Patient characteristics

During the study period, 748 patients were diagnosed with SBP. From these patients, 334 (44.7%) who were diagnosed with culture-positive SBP were enrolled in this study, of which 270 were males and 64 were females. Their ages ranged from 18 to 87 years and the mean age was 55.5 ± 10.4 years. Hepatitis B virus (145 patients, 43.4%) was the most common cause of cirrhosis, followed by alcohol (123 patients, 36.8%), and hepatitis C virus (31 patients, 9.3%). The patients were divided into two groups according to the type of infection: the nosocomial SBP group (*n* = 155, 46.4%) and the community-acquired SBP group (*n* = 179, 53.6%). There were no statistically significant differences in age, sex, liver cirrhosis cause, Child-Pugh classification, hepatic encephalopathy, septic shock or gastrointestinal bleeding between the two groups. Concerning clinical signs, such as fever, abdominal pain, renal failure and hepatocellular carcinoma were statistically significantly more common among the patients with nosocomial infections. The clinical and demographic characteristics of the study population are shown in Table 1.

**Table 1:** Baseline characteristics of patients with spontaneous bacterial peritonitis

Characteristic	Total (*n* = 334, %)	Nosocomial SBP (*n* = 155, %)	Community-acquired SBP (*n* = 179, %)	*P* value
Age, mean years ± SD	55.5 ± 10.4	56.3 ± 10.3	54.9 ± 10.4	0.222
Sex, Male	270 (80.8)	127 (81.9)	143 (79.9)	0.635
Main cause of liver cirrhosis (n, %)				0.903
HBV infection	145 (43.4)	67 (43.2)	78 (43.6)	0.949
HCV infection	31 (9.3)	17 (11.0)	14 (7.8)	0.323
Alcohol use	123 (36.8)	55 (35.5)	68 (38.0)	0.636
Primary biliary cirrhosis	15 (4.5)	7 (4.5)	8 (4.5)	0.984
Other	20 (6.0)	9 (5.8)	11 (6.1)	0.896
Child-Pugh classification				0.453
Class B	82 (24.6)	41 (26.5)	41 (22.9)	
Class C	252 (75.4)	114 (73.5)	138 (77.1)	
MELD score ± SD	16.9 ± 9.7	17.6 ± 10.0	16.0 ± 9.4	0.218
Initial presenting symptoms				
Fever	178 (53.3)	107 (69.0)	71 (39.7)	< 0.001
Vomiting/diarrhea	57 (17.1)	30 (19.4)	27 (15.1)	0.301
Abdominal pain	153 (45.8)	81 (52.3)	72 (40.2)	0.028
Hepatic encephalopathy	69 (20.7)	38 (24.5)	31 (17.3)	0.105
Gastrointestinal bleeding	38 (11.4)	18 (11.6)	20 (11.2)	0.900
Renal failure	65 (19.5)	40 (25.8)	25 (14.0)	0.006
Septic shock	25 (7.5)	13 (8.4)	12 (6.7)	0.560
Hepatocellular carcinoma	93 (28.7)	55 (35.5)	38 (21.2)	0.004

### Microbiological characteristics

A total of 334 pathogens were isolated from the ascetic fluid samples from the patients with SBP. There were 178 (53.3%) strains of Gram-negative bacteria, 138 (41.3%) strains of Gram-positive bacteria, 14 strains of fungi and 4 anaerobic strains. *E. coli* was the major pathogen (81 isolates, 24.3%), followed by *Klebsiella pneumoniae* (40 isolates, 12.0%) and *Enterococcus faecium* (35 isolates, 10.5%). As shown in Table 2, in the nosocomial group, the most common bacteria were *Enterococcus* (*n* = 43, 27.7%), followed by *E. coli* (*n* = 32, 20.6%), *K. pneumoniae* (*n* = 14, 9.0%), *coagulase-negative Staphylococci* (*n* = 12, 7.7%) and *S. aureus* (*n* = 9, 5.8%). Contrastingly, in the community-acquired group, the most common bacterial species were *E. coli* (*n* = 49, 27.4%) and *K. pneumoniae* (*n* = 26, 14.5%), followed by the *Streptococcus* genus (*n* = 23, 12.8%), *coagulase-negative Staphylococci* (*n* = 19, 10.6%) and the *Enterococcus* genus (*n* = 11, 6.1%). No statistically significant differences were found in the distribution of *E. coli*, *K. pneumoniae*, *coagulase-negative Staphylococcus*, and *S. aureus* between the two groups. However, there were more Gram-positive cocci in the nosocomial group (*n* = 73) than in the community-acquired group (*n* = 65, *P* < 0.05). Statistically significant differences were also found in the distribution of *Enterococci* between the two groups (6.1% vs. 27.7%, *P* < 0.001), and *Enterococci* were more commonly found in the nosocomial group.

**Table 2:** Bacteria distributions in nosocomial and community-acquired spontaneous bacterial peritonitis

Pathogen	No. (%) of patients			*p* value
	Total (n = 334, %)	Nosocomial SBP (n = 155, %)	Community-acquired SBP (n = 179, %)	
Gram-negative bacteria	178 (53.3)	75 (48.4)	103 (57.5)	0.094
*Escherichia coli*	81 (24.3)	32 (20.6)	49 (27.4)	0.152
*Klebsiella pneumoniae*	40 (12.0)	14 (9.0)	26 (14.5)	0.123
*Acinetobacter spp.*	11 (3.3)	6 (3.9)	5 (2.8)	0.582
*Enterobacter cloacae*	7 (2.1)	3 (1.9)	4 (2.2)	0.849
*Enterobacter species*	19 (5.7)	8 (5.2)	11 (6.1)	0.699
*Pseudomonas aeruginosa*	7 (2.1)	3 (1.9)	4 (2.2)	0.849
*Aeromonas species*	4 (1.2)	1 (0.6)	3 (1.7)	0.719
*Sphingomonas paucimobilis*	1 (0.3)	1 (0.6)	0 (0.0)	0.464
*Salmonella*	1 (0.3)	0 (0.0)	1 (0.6)	NS
*Stenotrophomonas maltophilia*	7 (2.1)	7 (4.5)	0 (0.0)	0.004
Gram-positive bacteria	138 (41.3)	73 (47.1)	65 (36.3)	0.046
*Streptococcus pneumoniae*	6 (1.8)	1 (0.6)	5 (2.8)	0.289
*Streptococcus (other than S. pneumoniae)*	26 (7.8)	8 (5.2)	18 (10.1)	0.096
*Enterococcus faecalis*	16 (4.8)	14 (9.0)	2 (1.1)	0.002
*Enterococcus faecium*	35 (10.5)	28 (18.1)	7 (3.9)	< 0.001
*Other Enterococcus*	3 (0.9)	1 (0.6)	2 (1.1)	NS
*Staphylococcus aureus*	16 (4.8)	9 (5.8)	7 (3.9)	0.418
*Coagulase-negative staphylococci*	31 (9.3)	12 (7.7)	19 (10.6)	0.376
*Listeria monocytogenes*	5 (1.5)	0 (0.0)	5 (2.8)	0.064
Fungi	14 (4.2)	6 (4.5)	8 (3.9)	0.786
*Candida albicans*	7 (2.1)	3 (2.6)	4 (1.7)	
*Candida glabrata*	3 (0.9)	1 (0.6)	2 (1.1)	
*Candida tropicalis*	2 (0.6)	1 (0.6)	1 (0.6)	
*Candida parapsilosis*	1 (0.3)	1 (0.6)	0 (0.0)	
*Cryptococcus neoformans*	1 (0.3)	0 (0.0)	1 (0.6)	
Anaerobe	4 (1.2)	1 (0.6)	3 (1.7)	0.627

The carbapenem antibiotics, amikacin and piperacillin/tazobactam, were the most consistently active in vitro drugs against *E. coli* and *K. pneumonia* in both the nosocomial and community-acquired infection groups (Table 3). Piperacillin/tazobactam and levofloxacin were significantly more effective against community-acquired *E. coli* infections, whereas cefepime was significantly more effective against community-acquired *K. pneumonia* infections (all *P* < 0.05). In addition, forty strains of extended-spectrum beta lactamase (ESBL)-producing *E. coli* and 6 strains of ESBL-producing *K. pneumonia* were detected, with detection rates of 49.4% (40/81) and 15.0% (6/40), respectively. The number of ESBL-producing *E. coli* was significantly higher for the nosocomial infections than for the community-acquired infections (*P* < 0.001). However, no obvious differences were observed for the *K. pneumonia* infections.

**Table 3:** Comparison of the drug resistance of major gram-negative bacteria to commonly used antibacterial agents between nosocomial and community-acquired SBP

Antibiotics	*Escherichia coli* (*n* = 81)			*Klebsiella pneumoniae* (*n* = 40)		
	Nosocomial (n = 32)	Community-acquired (n = 49)	*p value*	Nosocomial (n = 14)	Community-acquired (n = 26)	*p value*
ESBL	25 (78.1)	15 (30.6)	0.000	4 (28.6)	2 (7.7)	0.194
Ampicillin	29 (90.6)	43 (87.8)	0.968	14 (100)	24 (92.3)	0.533
Piperacillin	28 (87.5)	37 (75.5)	0.299	6 (42.9)	3 (11.5)	0.062
Amoxicillin-clavulanic acid	12 (37.5)	10 (20.4)	0.091	3 (21.4)	1 (3.8)	0.224
Piperacillin/tazobactam	4 (12.5)	0	0.022	2 (14.3)	0	0.117
Cefazolin	26 (81.2)	13 (26.5)	0.000	4 (28.6)	4 (15.4)	0.562
Ceftazidime	25 (78.1)	13 (26.5)	0.000	4 (28.6)	2 (7.7)	0.194
Cefotaxime	27 (84.3)	15 (30.6)	0.000	4 (28.6)	2 (7.7)	0.194
Cefepime	23 (71.9)	11 (22.4)	0.000	4 (28.6)	0	0.011
Aztreonam	18 (56.3)	10 (20.4)	0.001	2 (14.3)	1 (3.8)	0.571
Imipenem	2 (6.3)	0	0.153	2 (14.3)	0	0.117
Meropenem	1 (3.1)	0	0.395	1 (7.1)	0	0.350
Amikacin	2 (6.3)	0	0.153	1 (7.1)	0	0.350
Levofloxacin	23 (71.9)	21 (42.9)	0.01	3 (21.4)	1 (3.8)	0.224
SMZ	21 (65.6)	30 (61.2)	0.688	3 (21.4)	4 (15.4)	0.965
Gentamicin	17 (53.1)	25 (51.0)	0.853	2 (14.3)	2 (7.7)	0.912
Tetracycline	25 (78.1)	39 (79.6)	0.874	4 (28.6)	4 (15.4)	0.562

Vancomycin, linezolid and teicoplanin showed good antibacterial activities against *S. aureus* and *Enterococci* (Table 4). In addition, *S. aureus* displayed fairly high sensitivity (> 85%) to nitrofurantoin, rifampin and sulfamethoxazole, but the community-acquired isolates of *S. aureus* were more sensitive to clindamycin than the nosocomial-acquired isolates (*P* = 0.044). *Enterococci* susceptibility to erythromycin and nitrofurantoin in the community-acquired group was stronger than in the nosocomial isolates (all *P* < 0.05).

**Table 4:** Comparison of the drug resistance of major gram-positive bacteria to commonly used antibacterial agents between nosocomial and community-acquired SBP

Antibiotics	*Staphylococcus aureus* (*n* = 16)			*Enterococcus* (*n* = 54)		
	Nosocomial (n = 9)	Community-acquired (n = 7)	*p value*	Nosocomial (n = 43)	Community-acquired (n = 11)	*p value*
Penicillin	8 (88.9)	5 (71.4)	0.550	37 (86.0)	8 (72.7)	0.546
Oxacillin	6 (66.7)	3 (42.9)	0.615	–	–	–
Erythromycin	8 (88.9)	4 (57.1)	0.262	36 (83.7)	6 (54.5)	0.038
Clindamycin	7 (77.8)	1 (14.3)	0.041	–	–	–
Nitrofurantoin	0	0	NS	25 (58.1)	2 (18.2)	0.043
Rifampicin	0	0	NS	37 (86.0)	8 (72.7)	0.546
Tetracycline	4 (44.4)	2 (28.6)	0.633	24 (55.8)	6 (54.5)	0.940
Ciprofloxacin	5 (55.6)	2 (28.6)	0.358	32 (74.4)	8 (72.7)	0.909
SMZ	1 (11.1)	1 (14.3)	NS	–	–	–
Linezolid	0	0	NS	0	0	NS
Vancomycin	0	0	NS	8 (18.6)	1 (9.1)	0.762
Teicoplanin	0	0	NS	4 (9.3)	0	0.571

### Clinical outcome and predictors of mortality

The overall 30-day mortality rate for patients with SBP was 30.2% (101 of 334 cases). The mortality rate for nosocomial SBP was significantly higher than that for community-acquired SBP (36.8%, 57/155 cases vs. 24.6%, 44/179 cases; *P* = 0.016). The factors associated with the 30-day mortality rate are shown in Table 5. The multivariate analysis revealed that the Child-Pugh classification (*p* = 0.007; HR, 2.167; 95% CI,1.236–3.798), nosocomial infection (*p* = 0.044; HR,1.514; 95% CI,1.012–2.267), hepatocellular carcinoma (*p* = 0.002; HR, 1.930; 95% CI, 1.284–2.901), renal failure (*p* < 0.001; HR, 2.244; 95% CI, 1.440–3.496) and hepatic encephalopathy (*p* = 0.012; HR, 1.739; 95% CI, 1.129–2.677) were the independent predictors of 30-day mortality.

**Table 5:** Risk factors for 30-day mortality in patients with spontaneous bacterial peritonitis

Risk factor	Survivors (*n =* 233)	Nonsurvivors (*n* = 101)	Univariate	Multivariate		
			*P* value	*p* value	HR	95% CI
Age, mean years _ SD	55.3 ± 10.7	56.1 ± 9.9	0.869	–	–	–
Male	191	79	0.401	–	–	–
Child-Pugh classification			0.007	0.007	2.167	1.236–3.798
Class B	67	15				
Class C	166	86				
Hepatocellular carcinoma	53	40	0.005	0.002	1.930	1.284–2.901
Nosocomial infection	98	57	0.005	0.044	1.514	1.012–2.267
Resistance to third - generation cephalosporins	77	40	0.321	–	–	–
Gastrointestinal bleeding	23	15	0.105	–	–	–
Renal failure	34	31	0.001	< 0.001	2.244	1.440–3.496
Septic shock	14	11	0.156	–	–	–
Hepatic encephalopathy	37	32	< 0.001	0.012	1.739	1.129–2.677

## Discussion

SBP is a serious complication and common cause of death in patients with liver cirrhosis, but early diagnosis and the timely application of antibiotic therapy can significantly decrease its mortality rate [1, 2]. However, the etiological patterns of peritonitis show huge regional differences [4, 9, 10]. Thus, studying the epidemiology of SBP should be conducted at a local level with regard to the use of empirical therapy.

Gram-negative bacteria like *E. coli* and *K. pneumonia* are the most common causes of SBP [1, 2]. But several studies in recent years have shown that the major pathogens responsible for SBP have shifted from Gram-negative bacteria to Gram-positive cocci [14–17]. In contrast to these findings, and in agreement with the traditional model, our results show that Gram-negative bacteria (53.3%) remain the main etiological agents of SBP and that *E. coli* (24.3%) was its most common cause. Notably, Gram-negative bacteria were predominant in the community-acquired infections, whereas Gram-positive organisms were predominant in the nosocomial infections. This result is consistent with the data obtained from other investigations in mainland China [18, 19], indicating that no significant changes in the proportion of Gram-negative to Gram-positive infections occurred in the Chinese patients with SBP.

In recent years, some studies have reported on the emergence of resistance to TGCs in SBP [20–22]. Indeed, nosocomial infections are considered an independent predictor of resistance to these agents [4, 23]. In the present study, the resistant organisms cultured from ascites fluid mainly included *Enterococci*, ESBL-producing *Enterobacteriaceae* and some other naturally resistant bacteria (*Stenotrophomonas maltophilia* and *Listeria monocytogenes*). We found that ESBL production was higher in the samples from the nosocomial infection cases. Consequently, TGC resistance in the major Gram-negative bacteria was higher in the nosocomial group than in the community-acquired group. However, in a recent meta-analysis, Fiore et al found no significantly higher risk of TGC-resistant strains in nosocomial SBP compared with community-acquired SBP in China, although the total rate of TGC resistance was much higher than that seen in other countries [24]. This discrepancy may be partly explained by differences in the study populations. Beijing Youan Hospital is one of the largest liver disease treatment centers in China, with patients coming from all over the country. Some of these patients might have been undergoing antibiotic therapy in other hospitals. In addition, the *E. coli* strains obtained from the patients with nosocomial SBP showed much higher resistance to piperacillin/tazobactam (12.2% vs. 0.0%) and levofloxacin (71.9% vs. 42.9%), than those from patients with community-acquired SBP. *K. pneumoniae*, however, was more resistant to cefepime when it was isolated from the nosocomial cases than from the community-acquired cases (28.8% vs. 0.0%). There was also a significant difference in the distribution of *Enterococci* between the two groups (nosocomial, 6.1% vs. community-acquired, 27.7%). *Enterococci* are resistant to a variety of antibacterial agents, including TGC, but are highly sensitive to vancomycin, linezolid and teicoplanin. This suggests that when choosing the treatment for empiric therapy of patients with SBP medics should consider both the acquisition site of infection (nosocomial or community-acquired infection) and the local epidemiological situation. The new guidelines from EASL also mention another epidemiological entity; specifically, health-care associated SBP, which requires the same therapeutic approach as that used for nosocomial SBP [11]. However, as there is still controversy about its utility [25], health-care associated SBP was not divided into a separate group in the present study.

Continual progress in health technology has markedly improved the prognosis for cirrhotic patients with SBP. But the one-year survival rate after recovery from the first episode of SBP is only 30–40% [26, 27]. A number of studies have sought to identify prognostic factors in patients with SBP [4, 10, 12, 23, 28, 29]. Consistent with other studies [4, 28, 29], our results show that the Child-Pugh classification, concomitant hepatocellular carcinoma, renal failure presentation and hepatic encephalopathy are all significant risk factors for the 30-day mortality associated with SBP. As for nosocomial infections being a prognostic factor for SBP, some divergent opinions still exist. In our study, even after adjusting for the other prognostic factors associated with mortality, nosocomial SBP (*p* = 0.044; HR, 1.514; 95% CI, 1.012–2.267) was still identified as an independent risk factor for mortality. This view is consistent with the study findings of Cheong et al*,* [4] but differs from other studies in Korea [12, 13], which have all highlighted that the acquisition site of infection does not affect the clinical outcomes for patients with SBP. Differences in the study populations and variable therapeutic regimens may play a part in this discrepancy. But researchers also think that the high mortality rate for patients with SBP reflects both the presence of infectious diseases and the underlying illness itself. In general, patients with nosocomial SBP have more severe underlying illnesses than those with community-acquired SBP [14]; hence, it is not surprising that nosocomial SBP has a worse prognosis. Previous studies [4, 23, 28, 29] have also reported that resistance to TGC is an independent predictor of mortality in patients with SBP. However, the effects of such resistance could not be confirmed in our study. The difference between these other studies and our own may result from heterogeneity in the pathogens. Clearly, a bigger sample size will be needed in future research to verify this.

Our study has some limitations. First, the study comprised a single-center retrospective design and a relatively small sample size, and this may have compromised its statistical power. Second, the 2 groups are not completely comparable in their baseline characteristics (i.e., the higher prevalence of HCC in nosocomial infections reflect that these patients had a more advanced liver disease), to some extent, these selective bias could have influenced the higher mortality rate in the nosocomial SBP group. Third, only patients with SBP based on positive cultures were included in this study; we excluded patients with culture-negative neutrocytic ascites. Thus, selection bias may have occurred.

## Conclusion

In conclusion, in the present study, Gram-negative bacteria remain the most prevalent cause of SBP, but compared with the community-acquired group, the proportion of *Enterococci* responsible for SBP was significantly higher in the nosocomial group. The resistance rate of the main pathogenic bacteria to TGC was very high, particularly in patients with nosocomial SBP. Thus, the choice of antibiotics used as empirical therapy in patients with SBP should consider both the acquisition site of infection and the local epidemiological situation. Of particular note, nosocomial acquisition was found to be significantly associated with higher mortality rates, along with Child-Pugh classification, concomitant hepatocellular carcinoma, renal failure, and hepatic encephalopathy.

## Abbreviations

SBP: Spontaneous bacterial peritonitis
ESBL: Extended-spectrum beta lactamase
TGC: Third-generation cephalosporins
